# Supplementary material for: The Female-Biased General Odorant Binding Protein 2 of Semiothisa cinerearia Displays Binding Affinity for Biologically Active Host Plant Volatiles
Source: Biology (Basel). 2024 Apr 18;13(4):274. doi: 10.3390/biology13040274 (PMC11048283; doi:10.3390/biology13040274)
Supplement: Supplementary file 1 [file biology-13-00274-s001.zip › biology-2949489-supplementary.pdf]

Supplementary Information for

The female-biased general odorant binding protein 2 of *Semiothisa cinerearia* displays binding affinity to biologically active host plant volatiles

Jingjing Tu[1,2], Zehua Wang[1], Fan Yang[1], Han Liu[1], Guanghang Qiao[1], Aihuan Zhang[2] and Shanning Wang[1,*]

[1] Key Laboratory of Environment Friendly Management on Fruit and Vegetable Pests in North China (Coconstructed by the Ministry and Province), Ministry of Agriculture and Rural Affairs, Institute of Plant and Environment Protection, Beijing Academy of Agriculture and Forestry Sciences, Beijing 100097, China; 15910506265@163.com (J.T.); wangzehua200707@163.com (Z.W.); evelynyangfan@163.com (F.Y.); 15230451215@163.com (H.L.); qghang98@126.com (G.Q.)

[2] College of Bioscience and Resources Environment, Beijing University of Agriculture, Beijing 102206, China; zhangaihuan@126.com

* Correspondence: wangshanning@yeah.net

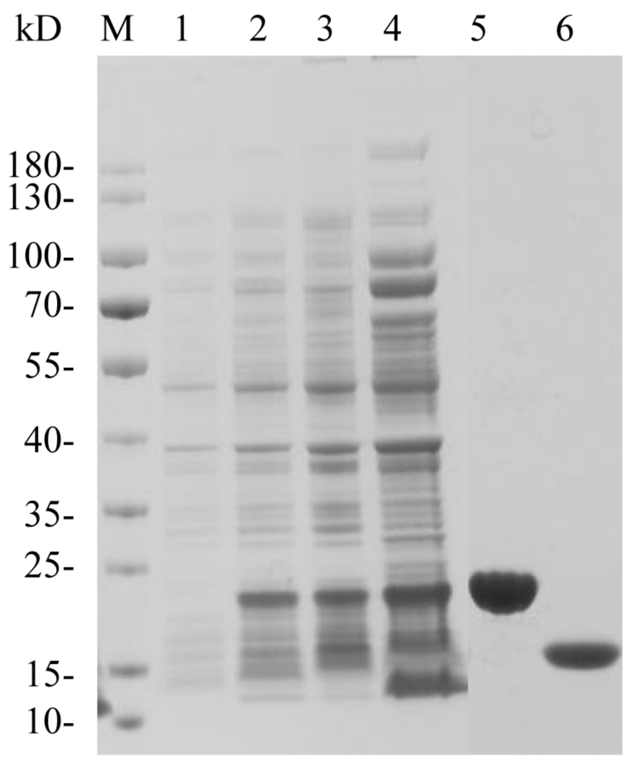

Figure S1. SDS-PAGE analysis of the recombinant ScinGOBP2. M: molecular weight markers, 1: cell pellet before induction with IPTG, 2: cell pellet after induction, 3: pellet after sonication, 4: supernatant after sonication, 5: protein purified by affinity chromatography, and 6: purified protein after digestion with enterokinase.

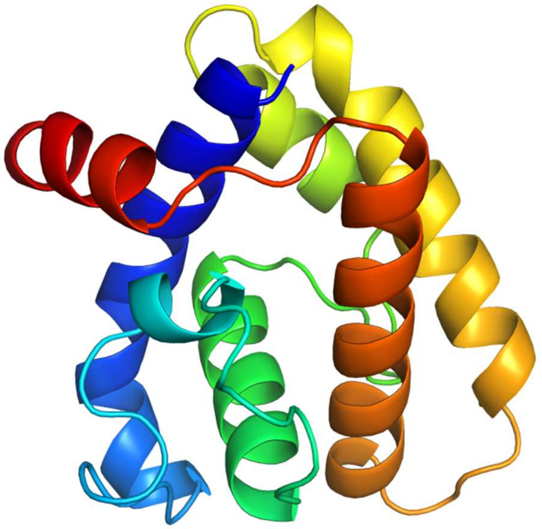

Figure S2. Three-dimensional (3D) structure of ScinGOBP2.

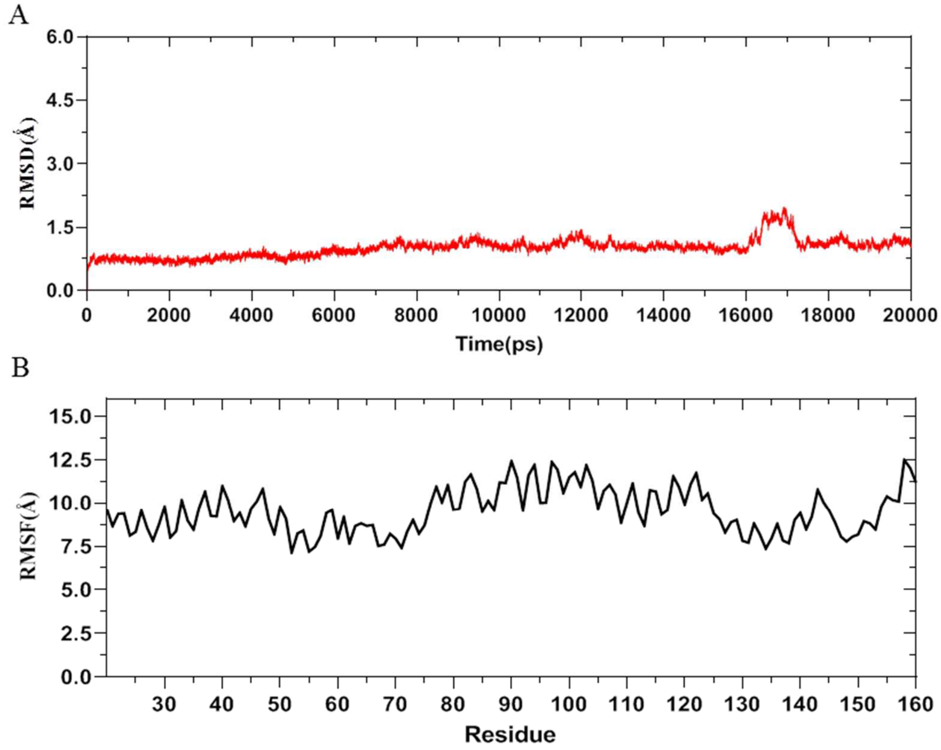

Figure S3. MD simulation results of ScinGOBP2. (A) RMSD plot showing the conformational changes of the protein till 20000 ps. (B) RMSF plot showing residue fluctuations for 20000 ps.

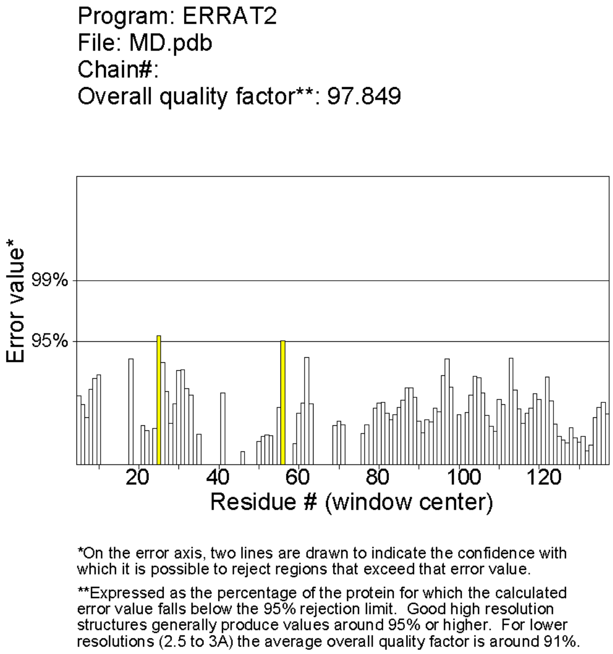

Figure S4. ERRAT results from the predicted 3D model of ScinGOBP2.

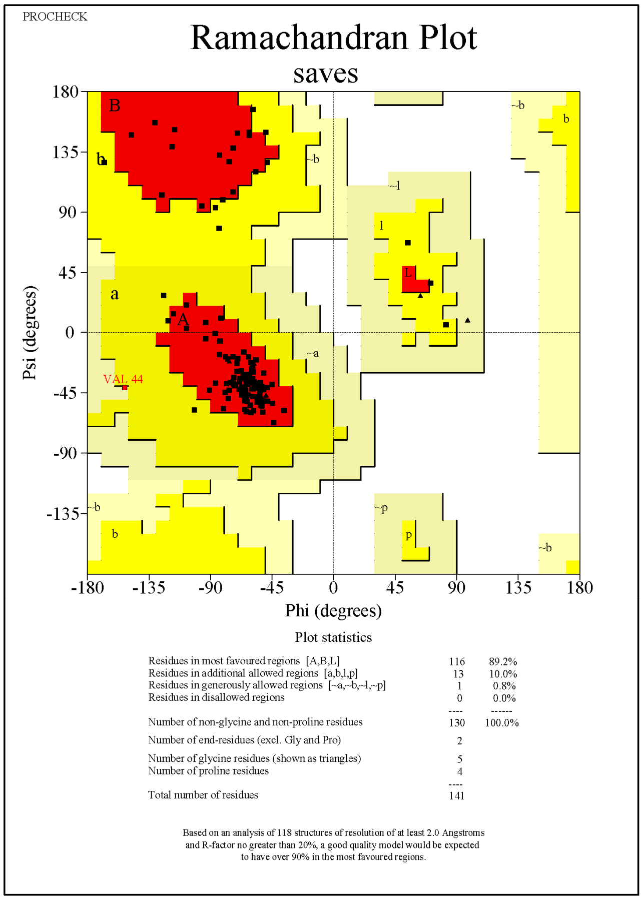

Figure S5. PROCHECK results from the predicted 3D model of ScinGOBP2.

Table S1. Primers used in this study.

Gene	Forward primer (5′-3′)	Reverse primer (5′-3′)
Gene cloning		
GOBP2	ATGTCAGAGCCGCTAGTGG	CTAATACTGTTCTAAAACAGC
RT-PCT		
GOBP2	TCCCCAAACATATTAGAAGAGTTCCA	TTCCAAAACAGCCTCAATCATGG
β-actin	CCTACAACTCCATCATGAAGTGTGA	GTATTCCTGTTTCGAGATCCACATC
qRT-PCT		
GOBP2	CTGTTGATGGTAGCGGTGGT	TGAGAGCCTTCCCAAAGTGC
β-actin	CAGAAGGACTCGTACGTGGG	GTAGAACGTGTGGTGCCAGA
TBP	CCACTACCTGCAGAACGTGT	GTTTGAGAGCGGGCAGTTTG
